# Utilization of Continuous Positive Airway Pressure (CPAP) by Emergency Medical Services: Updated Systematic Review and Meta-analysis

**DOI:** 10.31661/gmj.v12i.2957

**Published:** 2023-10-09

**Authors:** Roshan Dhakal, Deeven Karki, Sujha Ghimire, Rubiya Ali, Samia Dawach, Asra Iqbal, Roohie Farzaneh, Sara Rahsepar, Maryam Panahi, Farhad Bagherian, Behrang Rezvani Kakhki, Zahra Acheshmeh, Somayyeh Ahmadnezhad, Fatemeh Maleki, Uzair Yaqoob, Mohammad Zarenezhd

**Affiliations:** ^1^ Department of Medicine, Nepal Medical College, Kathmandu, Nepal; ^2^ Department of Medicine, Cleveland Clinic Foundation, Cleveland, USA; ^3^ Department of Medicine,The Indus Hospital, Karachi, Pakistan; ^4^ Department of Medicine, Bayview Hospital, Karachi, Pakistan; ^5^ Jinnah Postgraduate Medical Centre, Karachi, Pakistan; ^6^ Department of Emergency Medicine, Faculty of Medicine, Mashhad University of Medical Sciences, Mashhad, Iran; ^7^ Department of Dermatology, Mashhad University of Medical Sciences, Mashhad, Iran; ^8^ Department of Emergency Medicine, Babol University of Medical Sciences, Babol, Iran; ^9^ Department of Emergency Medicine, Mashhad University of Medical Sciences, Mashhad, Iran; ^10^ Ramsar Campus, Mazandaran University of Medical sciences, Ramsar, Iran; ^11^ Department of Emergency Medicine, Faculty of Medicine, Birjand University of Medical Sciences, Birjand, Iran; ^12^ Department of Neurosurgery, Dr. Ruth K. M. Pfau Civil Hospital, Karachi, Pakistan; ^13^ Legal Medicine Research Center, Legal Medicine Organization of Iran, Tehran, Iran

**Keywords:** Positive-Pressure Respiration, Critical Care, Emergency Medical Technicians, Metaa-analysis

## Abstract

Background: While new studies are being published on the prehospital continuous positive airway pressure (CPAP) application in patients with respiratory failure with conflicting results, previous meta-analyses are showing the benefits of CPAP in the prehospital transfer of patients with respiratory distress. Before the clinical application of high-level evidence, updated pooled estimates are needed based on the growing literature. This study aimed to compare prehospital CPAP with the usual standard oxygen therapy of respiratory failure patients. Materials and Methods: PRISMA guidelines served as the framework for this updated review study. It is an extension of a prior systematic review. We conducted comprehensive searches across several databases, including PubMed, Web of Science, Embase, and Scopus, focusing on randomized trials that juxtaposed pre-hospital CPAP application against standard care. Our primary interest was to assess the in-hospital mortality risks, and we employed random effect models to aggregate risk ratios from the selected studies. Results: Four articles were gathered based on the review of the updated literature (2013 to November 2022) in conjunction with the research incorporated in the preceding meta-analysis with a total number of 747 patients receiving prehospital CPAP with 101 events of in-hospital mortality. In the standard treatment control groups, there were 713 patients and 115 deaths occurred. Pooled mortality risk comparison between the group of prehospital CPAP and standard care patients had no statistically significant difference (P=0.16). There was no heterogenicity. A regression between the year of the studies and the effect size showed increased RR in new studies (P=0.017). Conclusion: Still more randomized trials are needed with higher sample sizes to conclude the lifesaving efficacy of the out-of-hospital CPAP.

## Introduction

Acute respiratory distress syndrome (ARDS) is a critical and potentially fatal condition in the lungs that causes hypoxemia and the inability of the lungs to function normally [1][2]. The increase of non-functional space in the lungs plays a big role in increasing the mortality of these patients. In other words, any amount of lung tissue that does not participate in gas exchange increases the mortality rate [3][4]. Supportive treatment in ARDS focuses on limiting further damage to the lungs with appropriate mechanical ventilation [5]. Based on the results obtained from the study of various articles, it can be said that early mechanical ventilation with a lung support approach has a definite effect in reducing the mortality caused by acute respiratory distress syndrome [6]. In patients who develop ARDS outside the hospital, the pre-hospital emergency plays the main role in the initial treatment until reaching the hospital [7][8]. The emergency medical system through various resources such as manpower, equipment, facilities, and various programs provides appropriate and timely emergency services, with the main goal of saving human life, disability, and death caused by diseases and injuries [9]. For this purpose, it is necessary to first identify the challenges and obstacles to decision-making in the scene of technicians and explain the process by which emergency medical technicians make decisions for patients and injured to identify the points that can be improved and the existing threats and opportunities [10]. However, we don’t have enough proof about using breathing-aiding machines in ambulances for ARDS patients. One common machine is called continuous positive airway pressure (CPAP). It’s usually used to help people with sleep apnea breathe better. CPAP machines increase air pressure in airways, making it easier to inhale. Some experts think it might be helpful for ARDS patients too [11]. A study conducted by Mal and their team used a systematic approach to review and analyze various research trials. These trials looked at how noninvasive positive pressure ventilation [NIPPV] performed outside of the hospital compared to the usual treatments for adults experiencing severe breathing difficulties. In total, they examined data from seven different studies. What they found was that NIPPV had a positive impact. Specifically, it led to lower mortality rates for patients who received this treatment outside of the hospital when compared to those who received the standard care. This suggests that NIPPV could be a valuable option for individuals dealing with severe respiratory distress in non-hospital settings [11]. However, despite these positive findings, it is important to acknowledge that the research on this topic is constantly evolving and new studies are being published with contradictory findings. To fully understand the potential benefits of CPAP in prehospital ARDS patients, it is essential to conduct updated pooled estimates based on the expanding body of literature. This will allow for a comprehensive analysis of all available evidence, including the new studies that have been published since the previous meta-analyses. By doing so, we can gain a more accurate understanding of the effectiveness of CPAP in out-of-hospital respiratory distress.

## Materials and Methods

To ensure a comprehensive and methodical examination of the subject matter, we have followed the PRISMA (Preferred Reporting Items for Systematic Reviews and Meta-Analyses) guidelines in conducting this comprehensively updated systematic review and meta-analysis [12]. While it was not formally registered in protocol registration databases, we meticulously conducted searches in Medline, Web of Science, Embase, and Scopus databases for articles concerning the prehospital application of CPAP in ARDS patients. We specifically sought clinical trials for inclusion, limiting our search to articles published from 2013 onwards. Information predating 2014 was extracted from previous systematic reviews and meta-analyses [13]. Our search strategy employed various keyword combinations, including "CPAP," "NIV," "Continuous positive airway pressure," "Non-invasive ventilation," "prehospital," "EMS," "Emergency services," "ARDS," "Acute respiratory failure," and "ARF." All articles discussing CPAP usage in prehospital emergency services within the timeframe spanning from 2013 to the conclusion of November 2022 were included in the study. The search query used in PubMed was "Controlled Clinical Trial (Publication Type) AND (Continuous positive airway pressure OR CPAP OR Non-invasive ventilation OR NIV) AND (Acute respiratory distress syndrome OR ARDS OR Acute respiratory failure OR ARF) AND (prehospital OR EMS OR Emergency services)."

Studies with other non-randomized designs, or studies whose data were not sufficient, or where there was no access to the required information, were not included. Also, the records of repeated searches were excluded from the study. Articles only published in English had the selection criteria for entering the study. Gray literature studies were not included in the study. Initially, two independent researchers meticulously compiled a comprehensive list of titles and abstracts encompassing all available articles within the aforementioned databases. This meticulous process aimed to discern and subsequently select pertinent titles autonomously. Subsequently, the researchers individually integrated related articles into the research workflow. Following this step, an inventory of abstracts was thoughtfully curated, enabling the elimination of extraneous articles through a thorough evaluation of their respective abstracts. The subsequent stage involved a comprehensive review of the entire texts of the selected articles. Each article underwent a meticulous assessment by two reviewers working independently. In cases where disparities in judgment emerged between the two researchers, a third reviewer was engaged to arbitrate and reach a consensus.

The data extraction stage was performed to collect the required data of the mortality rates in each group of the RCTs along with other baseline characteristics of the articles and participants. Data for each study included in the review was extracted using a standardized data extraction form that included the following variables: study ID, study design, emergency condition, etiology of ARDS, country, sample size (n), age, gender (female), CPAP/PEEP (cm H2O), and scene to ED time. Two independent researchers conducted the data collection and the final reports were compared. If the two researchers didn’t agree on the collected data, they asked a third person to help decide. To check how fair the study was, they used a special checklist from the Cochrane Collaboration [14].

Statistical Analysis

Mortality rates were extracted from each group of CPAP and standard care for estimation of the effect size. The pooled Risk Ratio (RR) of mortality was calculated based on the RRs of each study in a random effect model using the Mantel-Haenszel estimation method [15]. Heterogenicity was evaluated using the I2 and H2 statistics. A forest plot was drawn to visualize the effect size of each study and pool results with confidence intervals. Publication bias was assessed by Egger’s and Begg’s tests. As Egger’s was showing asymmetry of the funnel plot of the log of RRs; while Begg was showing its symmetry, Harbord regression was conducted that also showed significant asymmetry. The trim and fill method was applied in case of publication bias [15]. Sensitivity analysis was performed using a Leave-one-out meta-analysis [16]. All statistical analyses were conducted in STATA version 17 (StataCorp LLC, USA), considering P-value as the 0.05.

## Results

**Figure-1:**
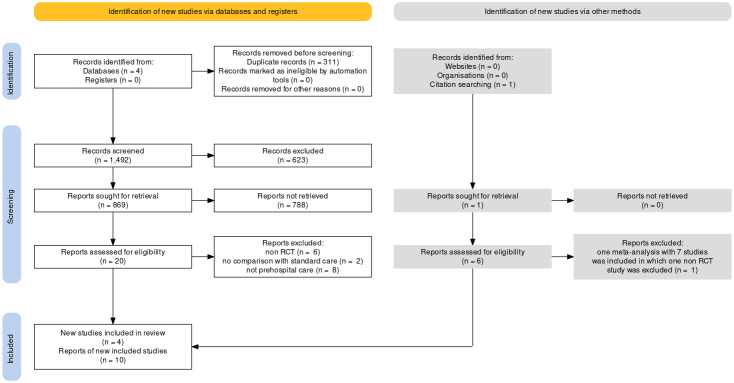
Figure-1 .Comprehensive PRISMA flowchart, providing a detailed visual representation of the study's progression and the systematic review process

**Table:** Table-1. Attributes of the Studies that Were Part of our Systematic Review and Meta-analysis

ID	study design	emergency condition	etiology of ARDS	country	n	age	gender (female)	CPAP PEEP, cm H2O	scene to ED time
Finn et al. [17]	RCT	acute respiratory distress	COPD, Heart failure, Influenza/Pneumonia, AMI, Other circulatory and respiratory disorders, Infectious, and other disease	Australia	708	77.3	311	5 to 10	37 vs. 35 min
Fuller et al. [18]	RCT	acute respiratory distress	COPD, Asthma, Heart failure, LRTI, PE	UK	77	71	29		43 vs. 36 min
Austin et al. [19]	RCT	severe acute cardiogenic pulmonary edema	Heart failure	Canada	50	79.8	27	10	35 vs. 36 in
Strnad et al. [20]	RCT	acute decompensated heart failure	Heart failure	Slovenia	20	81	12	5	NR

**Figure-2:**
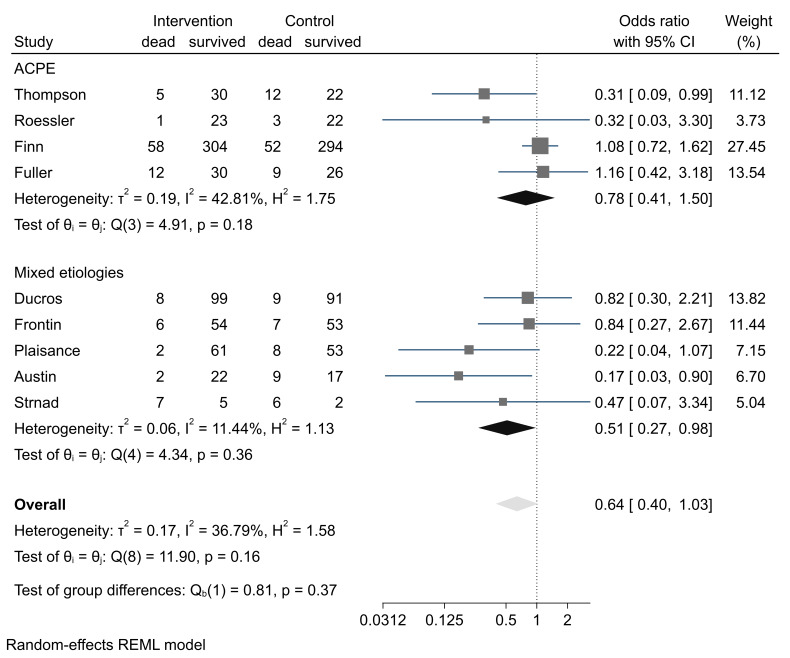
Figure-2 . Forest plot of the mortality risk ratio of the CPAP groups compared to the standard care.

**Figure-3:**
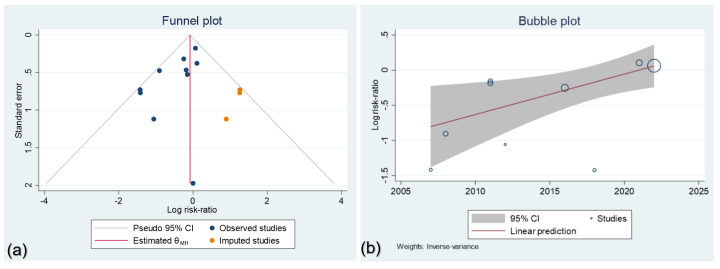
Figure-3. (a) Trim and filled funnel plot; yellow circles are imputed studies. (b) Meta-regression of risk ratio logarithm and year of publication.

**Figure-4:**
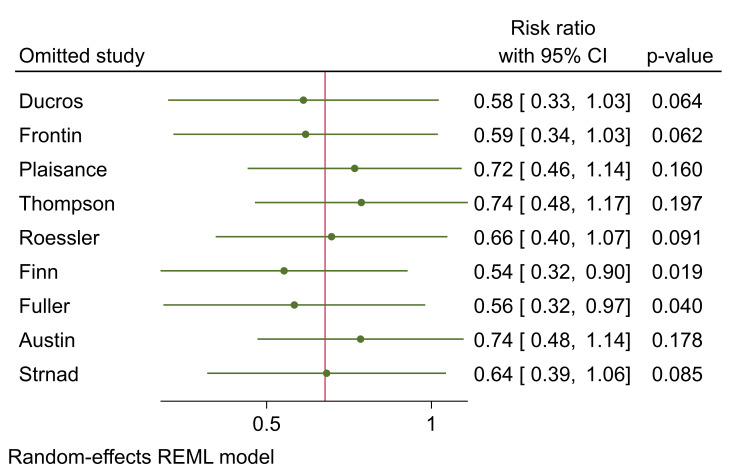
Figure-4. Forest plot of Leave-one-out sensitivity analysis

In the present study, 4 articles were part of the study due to the review of the updated literature (2014 to now) along with the studies included in the previous meta-analysis of Mal et al. From the search of 4 studied sources, 1803 initial cases were identified, after removing 311 duplicate cases, 1492 articles were examined in terms of titles, of which 623 cases Irrelevant were and deleted. Among the next 869 articles that were reviewed based on the article abstract, 788 unrelated items were removed. Finally, 20 studies were reviewed in the full text of which 4 did not have a randomized design and 12 were about CPAP application after hospitalization (Figure-1); finally, 4 RCTs were selected as shown in Table-1 [17][18][19][20].

Characteristics of Included Studies

Our study involved the analysis of 10 research studies, with some of them having been previously examined in a meta-analysis. For example, Ducros et al.’s study, which was part of a prior meta-analysis, focused on exploring the advantages of using CPAP for treating acute cardiogenic pulmonary edema (CPE) outside the hospital setting. They discovered that CPAP significantly improved early results when compared to solely receiving medical treatment. In fact, 79% of patients in the CPAP group experienced successful treatment, whereas only 63% in the control group did [21]. In another study by Frontin et al., the aim was to compare the effectiveness of conventional treatment for acute cardiogenic pulmonary edema (ACPE), both with and without CPAP, in patients outside the hospital. Interestingly, mortality rates and hospital stays were similar between groups [22]. Plaisance et al. found that early CPAP in ACPE, used alone or in combination with medical treatment in the late CPAP, improved clinical scores and arterial blood gases more effectively than medical treatment alone. Moreover, early CPAP significantly reduced the incidence of tracheal intubation and in-hospital mortality compared to the late CPAP group [23]. Roessler et al.’s study, study found that NIV was safe and effective for all patients, while controls did not work effectively for five out of 25 patients who eventually needed mechanical ventilation. Additionally, patients in the NIV group had fewer admissions to the ICU and shorter stays compared to those in the control group. Notably, NIV was more frequently used in hospitals for patients in the NIV group [24]. However, it’s important to note that CPAP is a type of NIV method, and this particular study may contribute to the variability in our findings when pooled with other studies. We chose not to include Schmidbauer et al.’s study [25] in our analysis because it did not report on mortality outcomes.

Thompson et al. found that using CPAP during pre-hospital care led to a significant 21% reduction in the mortality rate when compared to standard oxygen treatment [26]. In our systematic review, we included several new studies that provided differing results in certain instances. Strnad et al. carried out a study involving 20 patients with acute decompensated heart failure (ADHF), randomly assigning them to either a CPAP group or a control group. Lung ultrasound assessments were performed on both groups before and after treatment. Notably, the CPAP group displayed significant improvements in respiratory rate and arterial oxygen saturation [20]. Another study by Fuller et al. involved 77 patients, with 27.3% of them unfortunately passing away within 30 days [18]. Finn et al. conducted a study in which 708 patients were randomly assigned to either receive usual care or CPAP. The findings indicated that individuals in the CPAP group experienced a greater reduction in dyspnea scores and respiratory rate [17]. Additionally, Austin et al.’s pre-hospital randomized trial in Tasmania, Australia, involving 50 participants who had experienced a sudden onset of severe respiratory distress, revealed that the use of CPAP in combination with low-flow oxygen was associated with lower mortality rates, improved respiratory outcomes, and shorter hospital stays in comparison to standard oxygen therapy [19].

Results of Syntheses

Considering the studies included in the Mal et al. study, there was a total number of 747 patients receiving prehospital CPAP with 101 events of in-hospital mortality (Figure-2). In the standard treatment control groups, there were 713 patients and 115 deaths occurred. In the random effect model, pooled mortality risks had no statistically significant difference between the group of the CPAP and standard care patients (P=0.07). There was no heterogenicity (I2=36.79%). For adjusting the results based on the etiology of ARDS, individual patient data or adjusted risk ratios were not available for most studies. So, we performed a subgroup analysis based on the etiology of the ARF. In a subgroup of patients with ACPE, 4 studies were included and it was found that under a minor heterogeneity between studies (I2=42.81%), there was no statistical significance in case of risk of death between intervention and control subjects in a random effect model; RR was 0.78, with a 95% confidence interval ranging from 0.41 to 1.51; But, studies with mixed populations of ACPE and other pulmonary etiologies of ARF, like asthma, COPD, and pneumonia, pooled RR of 5 studies by random effect model revealed statistically significant lower risk of death in cases receiving prehospital CPAP intervention compared to controls [RR=0.51, 95%CI (0.27 to 98)].

The funnel plot was visualized based on the standard error of the logarithm of the RRs. There was a significant asymmetry in the funnel plot. Begg’s (P=0.28) showed its symmetry, while Harbord’s (P=009) and Egger’s (P=0.022) regression were showing significant asymmetry. A trim fill method was conducted and 3 studies were imputed in our included studies to omit the potential publication bias that was successful (Figure-3, a). However no changes happened in the final comparison of the mortality pooled risk between the study groups, RR=0.922, 95%CI of 0.73 to 1.16. A regression between the year of the studies and the effect size showed increased RR in new studies (P=0.017), as shown in Figure-3, b.

In a sensitivity analysis, we deleted the Strand et al. study as it had excluded critically ill patients. The mentioned study was not an RCT of CPAP intervention and it was an observational investigation examining the effectiveness of bedside ultrasonography.

After excluding that, both comparisons of sole ACPE patients and mixed etiology groups were non-significant, with risk ratios of 0.78 95%CI (0.47 to 1.51) and 0.49 95%CI (0.23 to 1.06). We further performed a leave-one-out meta-analysis. Figure-4 shows the final RR after excluding each study. Excluding the Finn and Fuller et al. studies, the results turned statistically significant. This finding is consistent with the results of the meta-regression as newer studies, Finn et al. in 2021 and Fuller et al. in 2022, are in contrast with previous older studies.

While we used mortality data in the Finn et al. study, mortality was a secondary outcome in their study, and the study was not powered for mortality. Their primary outcome was dyspnea scores which they think is more appropriate for the EMS studies and they stated that a higher number of patients is needed for mortality assessment. So, while this study has good quality, its power on the outcome of mortality is low. ACTIVE trial pilot results, having high quality had a low number of patients for 30-day intubation and mortality as it was piloted as well as the Strnad et al. that had a lower quality of evidence (Table-2).

## Discussion

**Table:** Table2. Evaluation of Study Quality Using the Cochrane Collaboration’s Assessment Tool

	Selection bias		Performance bias	Detection bias	Attrition bias	Reporting bias
ID	Random sequence generation	Allocation concealment	Blinding of participants and personnel	Blinding of outcome assessment	Incomplete outcome data	Selective reporting
Finn et al.[17]	↓	↓	↑	↓	↓	↓
Fuller et al.[18]	↓	↓	↑	↓	↓	↓
Austin et al.[19]	↓	↓	↑	~	↓	↓
Strnad et al.[20]	↓	↑	↑	~	↓	↓

**↑:** high bias possibility; **↓:** low bias possibility; **~:** unclear bis

Our Systematic review and meta-analysis study entered four new studies to previously conducted meta-analyses on the comparison of the mortality risk in prehospital CPAP and standard care. In conflict with the previous report (Mal et al.), we found that the pooled mortality risk difference between the group of prehospital CPAP and standard care patients had no statistically significant difference (P=0.11). Our regression model with the year of the study confirms this issue with the finding that the RR of the mortality has tended to 1 passing the time of publication. This means that the odds of mortality were different in old studies, while new studies do not show such differences among the groups of pre-hospital CPAP and standard care. The difference is due to new high-quality studies like Fuller et al. and Finn et al. studies that do not support CPAP’s effect on mortality reduction.

As well as our study, Mal et al. [12] study did not find any heterogenicity between the studies. While we only were able to assess the mortality as the outcome, Mal et al. also evaluated the intubation risk but new studies have not reported the required data for such analysis. As well as our study, Mal et al. mentioned the high potentiality of publication bias. There seem to be some studies with negative results that are not published or found by our and Mal’s search strategy. While they did not make any effort to adjust the results for the publication bias, we performed a trim-and-fill analysis. This analysis introduced 3 hypothetical studies as non-published studies but the final results did not change and there were no significant differences in the risk of death between the groups.

There were few previous systematic review studies published on this subject, before 2014. Applying critical appraisal of these studies, the chief focus is on the intervention of the prehospital CPAP and literature has not yet provided undeviating etiology of the ARDS, till 2014 [27][28][29][30]. The research design of most studies published till 2014 is not randomized and vast discrepancy in settings has limited the ability for decision-making for clinical practice [28]. The most powerful previous systematic review conducted by Goodacre et al. has limited their review to RCTs and Quasi-RCTs and also collected individual patient data that showed promising effects of the CPAP in reducing mortality with a good overall quality of the evidence [29]. With a similar and even higher quality of evidence, our study had different conclusions. Williams et al. study which also included two non-randomized trials had publication bias and they didn’t make any efforts to address the publication bias [24]. In a separate review conducted in 2015, CPAP was determined to be the most successful intervention in reducing death rate and mechanical ventilation when compared to standard treatment strategy. However, the proficiency of BiPAP remained uncertain. Interestingly, this review also identified that gender played a noteworthy role as a treatment effect modifier for mortality [30]. As a limitation, we were unable to use adjusted RR because the studies included in our meta-analysis did not perform any justifications on RR, and all crude RRs were reported. We are aware that crude RR is not as reliable as adjusted RR as it may be affected by confounding factors that could lead to inaccurate results. However, we did acknowledge this limitation in the study.

## Conclusion

We found that the pooled mortality risk difference between the group of prehospital CPAP and standard care patients had no statistically significant difference. More RCTs are needed with higher sample sizes to conclude the lifesaving efficacy of the CPAP.

## Conflict of Interests

None.
